# Mitigative Potential of Novel *Lactobacillus plantarum* TISTR 2076 against the Aflatoxins-Associated Oxidative Stress and Histopathological Alterations in Liver and Kidney of Broiler Chicks during the Entire Growth Period

**DOI:** 10.3390/toxins14100689

**Published:** 2022-10-08

**Authors:** Ashiq Ali, Aisha Khatoon, Hailah M. Almohaimeed, Faisal Al-Sarraj, Raed Albiheyri, Ibrahim Alotibi, Zain Ul Abidin

**Affiliations:** 1 Faculty of Agriculture and Veterinary Sciences, Superior University, Lahore 54000, Pakistan; 2 Department of Pathology, University of Agriculture Faisalabad, Faisalabad 38000, Pakistan; 3 Department of Basic Science, College of Medicine, Princess Nourah bint Abdulrahman University, Riyadh 11671, Saudi Arabia; 4 Department of Biological Science, Faculty of Science, King Abdulaziz University, Jeddah 21589, Saudi Arabia; 5 Centre of Excellence in Bionanoscience Research, King Abdulaziz University, Jeddah 21589, Saudi Arabia; 6 Health Information Technology Department, Applied College, King Abdulaziz University, Jeddah 22254, Saudi Arabia; 7 Veterinary Research Institute Lahore Cannt, Lahore 54000, Pakistan

**Keywords:** aflatoxins, *Lactobacillus plantarum*, amelioration, broiler, hematological, serum biochemical

## Abstract

Aflatoxins are the secondary metabolites produced by *Aspergillus flavus* and *Aspergillus parasiticus* and have severe pathological effects on the health of human and animals. The present study was designed to investigate the toxicopathological changes induced by aflatoxins and mitigative potential of *Lactobacillus plantarum* in broiler birds. One hundred and eighty broiler chicks at one day of age was procured from the local market, and chicks were equally divided into six groups with thirty birds in each group. These birds were treated with aflatoxins (300 and 600 µg/kg) and *Lactobacillus plantarum* (1 × 10^8^ cfu/kg of feed) in different combinations. The first group was kept as the control, and only a basal diet was provided to birds (BD). In the second group (AF1), the first level of aflatoxins (300 µg/kg) was fed to the birds. In the third group (AF2), the second level of aflatoxins (600 µg/kg) was fed to birds. In the fourth group (AF1LP), *Lactobacillus plantarum* was given with first level of aflatoxins. In the fifth group (AF2LP), *Lactobacillus plantarum* was given with the second level of aflatoxins, and in the 6th group (BDLP), *Lactobacillus plantarum* alone was fed to the chicks. This experimental study was continued for 42 days. Birds were slaughtered after 42 days, and different parameters were assessed. Parameters studied were gain in body weight, organ weight along with some histopathological, hematological, biochemical parameters and residues of aflatoxins in liver and kidney. *Lactobacillus plantarum* improved the body weight gain and restored the relative organ weight. Hepatic and renal biomarkers returned to normal concentrations, serum proteins were restored in combination group AF1LP, and partial amelioration was observed in the AF2LP group. Red blood cells, white blood cells, hemoglobin centration and packed cell volume became normalized in the AF1LP group, while partial amelioration was observed in the AF2LP group. LP also reduced the concentration of aflatoxin residues in liver kidney and improved the TAC concentrations. The results of this study elucidated the mitigative potential of *Lactobacillus plantarum* against serum biochemical, histopathological, hematological and toxicopathological changes induced by aflatoxins in the chicks.

## 1. Introduction

Aflatoxins are mainly produced by *Aspergillus flavus* and *Aspergillus parasiticus*, under favorable conditions, and natural substances promote the growth of these fungi [1,2]. Therefore, aflatoxins are mainly produced on commonly used foods and feeds [3]. The most toxic form of aflatoxins is aflatoxin B1, and it has carcinogenic effects in humans and animals. When animals are exposed to aflatoxins, it leads to a condition termed as aflatoxicosis, which causes pathological alterations in birds and animals [4,5]. Aflatoxicosis also causes severe economic losses along with health problems. These economic losses include low growth in livestock and poultry breeding units along with high mortality [6,7,8].

The organ most commonly affected by aflatoxins is liver. Aflatoxins increase the weight of liver, which disturbs the functions of this organ. Aflatoxins cause an increase in the level of aspartate aminotransferase and alanine aminotransferase, while decreasing the level of proteins, triglycerides, albumin, globulin and cholesterol in the blood, which decreases production in poultry [9]. In the kidney of poultry, aflatoxins enhance the apoptosis, while an increase in the size of kidney also occurs. Aflatoxins increase the level of creatinine and uric acid in the poultry blood [10]. 

Previous data are available for the prevention of aflatoxins in poultry feed using physical and chemical methods. However, the feasibility and safety of these methods at a large scale is a problem which is currently being debated. For example, the use of chemicals for aflatoxin destruction in grains and feed is unsafe because of residues remaining in the grains and feed [11]. Many aflatoxin binding agents have been used to remove the aflatoxins from the intestine into the feces to reduce the aflatoxin toxicity, but these binding agents also remove some essential nutrients from the gastro intestinal tract [10]. 

The use of beneficial microorganisms with probiotic properties proved helpful with many aspects. Useful aspects of probiotic bacteria include promotion of gut defense barrier and restoration of GUT microbiota [12]. Probiotics are well known for their antioxidant property and immunomodulatory action [13]. 

These beneficial microorganisms can be given to the poultry birds in the feed either single or in the mixture of useful microorganisms.

The use of lactic acid bacteria to control aflatoxins has been reported in few experimental studies. However, previous studies have elucidated the influence on very few parameters; therefore, this is a comprehensive study to explore the protective effect of *Lactobacillus plantarum* in broilers against aflatoxins. 

*Lactobacillus plantarum* (LP) is a Gram-positive bacterium with rod-shaped cells and occurs in chains or pairs. It grows at pH 3.2 or higher and at a temperature between 15 °C and 45 °C. Initially, this bacterium was isolated from saliva, but it is also found in the plant matter of anaerobic origin and fermented foods. This bacterium can adsorb mycotoxins in vitro, as reported by some researchers [14]. LP has the two basic mechanisms for the detoxification of aflatoxins, i.e., adsorption and biodegradation of aflatoxins into less toxic compounds. 

The previously reported literature is silent about the ameliorative effect of LP upon the gross and microscopic lesions of liver and kidney, serum biochemical and hematological alterations induced by aflatoxins in chickens. So, the present study was conducted to check the ameliorative potential of LP against aflatoxicosis in chickens. 

## 2. Results

### 2.1. Feed Intake

The feed intake of the broilers have been presented in Table 1. During the 1st week, all the groups showed non-significant differences compared to the control. Feed intake of AF1, AF2 and AF2LP was significantly lower as compared to the control, while groups AF1LP and LP showed non-significant differences. From the 3rd to 6th week, feed groups AF1, AF2 and AF2LP showed significantly lower feed intake, while AF1LP was non-significant compared to the control. LP group showed significantly higher feed intake from the 3rd to 6th week. From the 3rd to 6th week, the feed intake of AF1LP was significantly higher when compared with AF1 group. AF2LP showed significantly higher feed intake when compared with AF2. 

### 2.2. Body Weight Gain and Organ Weight

Table 2 and Table 3 show body weight and organ weight of all the groups. The initial body weights of chicks were between 39 g and 51 g. Body weight gain was higher in the BDLP group throughout the experiment, while significantly lower body weight gain was observed in AF1, AF2 along with group AF2LP. The body weight of group AF1LP was non-significant when compared with the control throughout the experiment. The relative weight of liver in groups AF1, AF2 and AF2LP were significantly lower, while groups AF1LP and BDLP were non-significant to the control. Relative weights of kidney showed same trend as in liver. From 3rd to 6th week the body weight of the group AF1LP was significantly higher when compared with AF1 group. AF2LP group also showed higher body weight when compared with the AF2 group, in which the second aflatoxins level was fed to the birds. 

### 2.3. Hematological Examination

**Figure 1:**
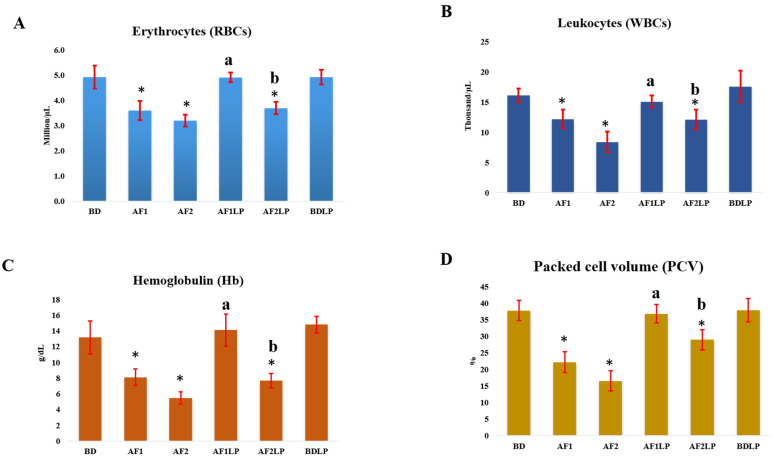
Hematological parameters of the birds fed aflatoxins alone and in combination with *Lactobacillus plantarum* RBCs count (**A**), WBCs (**B**), Hb concentration (**C**), and PCV% (**D**). Vertical error bars with stars are significantly different from the control. Vertical error bars with letters a and b are significantly different from AF1 and AF2 respectively. **Treatments:** BD = Basal diet; AF1 = Aflatoxins 300 µg/kg of feed; AF2 = Aflatoxins 600 µg/kg of feed; AF1LP = Aflatoxins 300 µg/kg of feed + *Lactobacillus plantarum* 1 × 10^8^ CFU; AF2LP = Aflatoxins 600 µg/kg of feed + *Lactobacillus plantarum* 1 × 10^8^ CFU; BDLP = Basal diet + *Lactobacillus plantarum* 1 × 10^8^ CFU.

Figure 1 presents the RBCs count, WBCs count, Hb concentration and hematocrit percentage (PCV) of all the groups.

Mean RBCs count, Hb concentration, and PCV percentage were higher in the BDLP group than the control. The mean RBCs count of AF1, AF2 and AF2LP was 3.2, 3.6 and 3.4 billion/µL, respectively, which was significantly less than the control, while the mean erythrocyte count in AF1LP was 4.9, which was non-significant when compared with the control. Hemoglobin concentration and hematocrit percentage also showed same trends. The average hemoglobin concentration in the AF1 and AF2 groups was 8.1 and 8.5 g/dL, respectively, which was lower than the control, while the hemoglobin concentration in AF1LP was 12.9 g/dL, which showed a non-significant difference with the control. The mean Hb concentration in the AF2LP was 7.7 g/dL, which was significantly lower when compared with the control. The packed cell volume of the AF1, AF2 and AF2LP groups was 22.2 and 16.6 and 29.0 percent, respectively, which was significantly lower than the control. The PCV percentage in the AF1LP was 36.8, which was non-significant compared with the control. The leukocytic count of AF1 and AF2 along with AF2LP were significantly lower, while that of groups AF1LP and LP were non-significant as compared to the control. The mean values of leukocyte count in the AF1, AF2 and AF2LP groups were 12.2 and 8.4 and 11.8 million/µL, respectively, which was significantly lower as compared to the control. The mean leukocyte count in the AF1LP was 15.1 million/µL, which significantly lower as compared to the control. The RBCs, WBCs counts, Hb concentration and PCV percentage were significantly higher in the AF1LP and AF2LP groups when compared with individual AF1 and AF2 groups, respectively. 

### 2.4. Serum Chemistry

**Figure 2:**
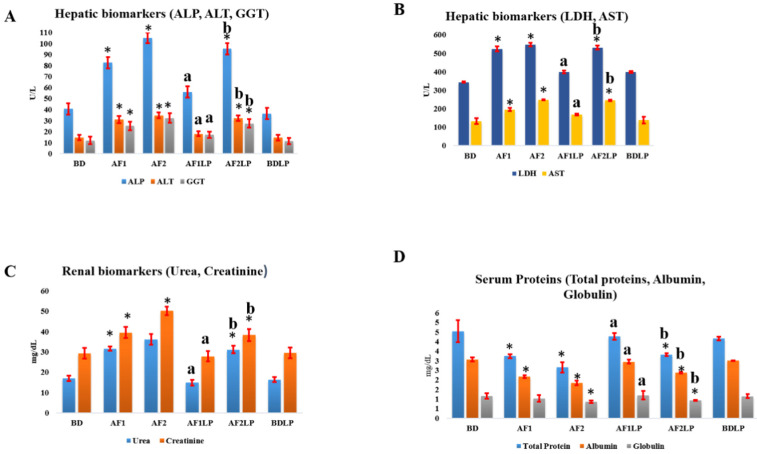
Serum biochemical parameters of the birds fed aflatoxins alone and in combination with *Lactobacillus plantarum*. Hepatic biomarkers (**A**,**B**), renal biomarkers (**C**), and Serum proteins (**D**). Vertical error bars with stars are significantly different from control. Vertical error bars with letters a and b are significantly different from AF1 and AF2 respectively. **Treatments:** BD = Basal diet; AF1 = Aflatoxins 300 µg/kg of feed; AF2 = Aflatoxins 600 µg/kg of feed; AFILP = Aflatoxins 300 µg/kg of feed + *Lactobacillus plantarum* 1 × 10^8^ CFU; AF2LP = Aflatoxins 600 µg/kg of feed + *Lactobacillus plantarum* 1 × 10^8^ CFU; BDLP = Basal diet + *Lactobacillus plantarum* 1 × 10^8^ CFU.

Figure 2 presents the mean concentrations of serum proteins, hepatic and renal biomarkers.

#### 2.4.1. Total Proteins, Albumin and Globulin

The serum concentrations of total protein, albumin and globulin are presented in Figure 2D. 

Serum proteins of groups AF1LP and BDLP were non-significant, while group AF1, AF2 and AF2LP showed significantly lower mean concentrations than the control. Serum albumin values of groups AF1 and AF2 showed significantly lower mean concentrations, while that of groups AF1LP and BDLP were non-significant as compared to the control. A similar pattern was observed in serum globulin values. The mean concentration of total proteins in the AF1, AF2 and AF2LP groups were 3.25, 2.66 and 3.32 mg/dL, which were lower than the control, while the AF1LP and BDLP showed 4.28 and 4.35 mg/dL, respectively, which showed no significant difference to the control. The chickens of the aflatoxins-intoxicated groups AF1 and AF2 showed 2.18 and 1.85 mg/dL of albumins, respectively, values which were significantly lower than the control. Among the probiotic-supplemented groups, AF1LP (2.95) was not significant with the control while AF2LP (2.28) showed significantly lower concentration of albumin. Serum globulin concentrations of all the groups were non-significant as compared to the control. Serum proteins concentrations of AF1LP and AF2LP groups were significantly higher as compared to AF1 and AF2 groups, respectively. 

#### 2.4.2. Hepatic Biomarkers

The serum concentration hepatic biomarkers has been presented in Figure 2A,B.

Serum AST, ALT, GGT, LDH and ALP values of groups AF1LP and BDLP were non-significant, while that of groups AF1 and AF2 along with the group AF2LP were significantly higher than the control (BD). 

The mean concentrations of serum ALP in AF1, AF2 and AF2LP were 82.6 U/L, 105.1 U/L and 95.2 U/L, respectively, which were higher than the control, while among the *Lactobacillus plantarum*-supplemented groups AF1LP and BDLP showed a non-significant difference with the control. Serum ALT concentrations of AF1 and AF2 groups were 30.9 U/L and 34.8 U/L, which were significantly higher than that of the control. Among the probiotic-supplemented groups, AF2LP showed a significantly higher value of serum ALT (30.0 U/L), while the AF1LP showed 18.0 U/L, which was non-significant when compared to the control group. Gamma glutamyl serum concentrations were significantly higher in the AF1, AF2 and AF2LP groups (25.1 U/L, 32.4 U/L and 27.4 U/L), respectively. The first probiotic-supplemented group AF1LP showed a non-significant difference with the control. The mean serum concentrations of LDH (524.1, 546.7 and 531.5) and AST (196.2, 248.0 and 245.7) of AF1, AF2 and AF2LP groups were higher than the control. The mean values of LDH (399.1) and AST (139.6) in the AF1LP group were non-significant when compared with the control. The hepatic biomarkers were significantly decreased in AF1LP and AF2LP when compared with the AF1 and AF2 groups, respectively.

#### 2.4.3. Renal Biomarkers

The serum concentration of renal biomarkers has been presented in Figure 2C. 

The concentrations of urea and creatinine were significantly higher in groups AF1, AF2 and AF2LP as compared to the control, while groups AF1LP and BDLP showed a non-significant difference to the control. Serum urea concentration in the AF1 and AF2 groups was 31.52 mg/dL and 36.17 mg/dL, respectively, which was significantly higher than the control, while the probiotic-supplemented groups AF1LP and AF2LP showed 14.89 mg/dL and 31.14 mg/dL, respectively; AF1LP was non-significant when compared with the control, while AF2LP showed a significantly higher value. Similar trends were observed in the serum concentrations of creatinine as in urea. Creatinine concentrations in the AF and AF2 groups were 39.45 mg/dL and 50.21 mg/dL, respectively, while in the probiotic-supplemented groups, these concentrations were 27.77 mg/dL and 38.28 mg/dL, respectively. AF1, AF2 and AF2LP showed significantly higher values, while AF1LP was non-significant when compared to the control. In the combination groups (AF1LP and AF2LP), the concentration of urea and creatine were significantly lower as compared to individual aflatoxins groups (AF1 and AF2).

#### 2.4.4. Serum TAC (Total Antioxidant Capacity) and TOS (Total Oxidant Status)

**Figure 3:**
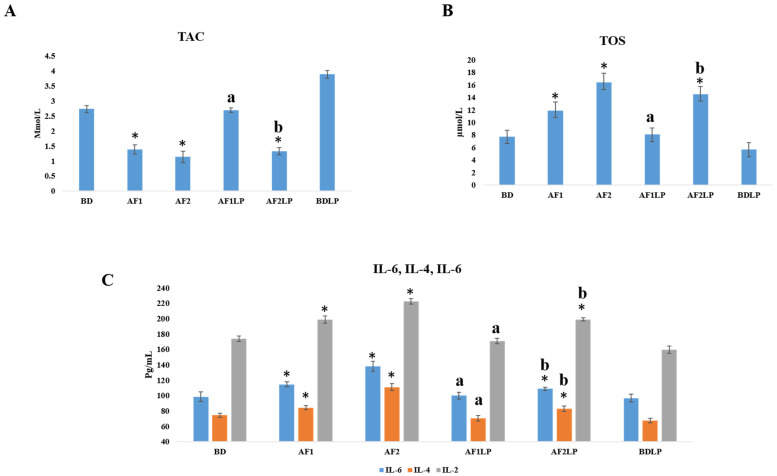
Serum TAC concentrations (**A**), serum TOS concentrations (**B**) and serum interleukin concentrations (**C**). Vertical error bars with stars are significantly different from control. Vertical error bars with letters a and b are significantly different from AF1 and AF2 respectively. **Treatments:** BD = Basal diet; AF1 = Aflatoxins 300 µg/kg of feed; AF2 = Aflatoxins 600 µg/kg of feed; AF1LP = Aflatoxins 300 µg/kg of feed + *Lactobacillus plantarum* 1 × 10^8^ CFU; AF2LP = Aflatoxins 600 µg/kg of feed + *Lactobacillus plantarum* 1 × 10^8^ CFU; BDLP = Basal diet + *Lactobacillus plantarum* 1 × 10^8^ CFU.

Serum TAC and TOS concentrations are presented in Figure 3A and 3B, respectively.

TAC values in the BDLP group were higher than the control, while the AF1LP was non-significant when compared with the control. The mean concentrations of TAC in the AF1 and AF2 groups were 1.39 mmol/L and 1.14 mmol/L, respectively, which were significantly lower than the control, while in the combination group AF1LP, the total antioxidant value was 2.69 mmol/L, which was nearly equal to normal. Supplementation of *Lactobacillus plantarum* in the AF2LP group did not completely ameliorate the toxic effects of aflatoxins. The mean concentration of TOS in the AF1LP was 8.07 µmol/L, which was non-significant when compared with the control, while the BDLP group showed significantly lower TOS values (5.65 µmol/L) as compared to the control. The average concentrations of TOS in the AF1 and AF2 groups were 11.91 µmol/L and 14.55 µmol/L, respectively, which were significantly high as compared to the control. The serum TAC concentration was significantly higher in the AF1LP group when compared with the AF1 group, while the AF2LP group showed a non-significant difference when compared to the AF2 group. The serum TOS concentration of the groups AF1LP and AF2LP were lower as compared to the AF1 and AF2 groups. 

#### 2.4.5. Serum Interleukins

Serum interleukins concentrations are presented in Figure 3C.

The concentrations of IL-2, IL-4 and IL-6 were normal in the control group (BD) and probiotic-supplemented group (BDLP). Significantly high concentrations of IL-2, IL-4 and IL-6 were observed in the AF1 and AF2 groups. The mean values of IL-2, IL-4 and IL-6 in chickens of the AF1 group were 198.66 pg/mL, 84.33 pg/mL, 114.66 pg/mL, respectively, while the mean values of IL-2, IL-4 and IL-6 in the AF2 group were 222.66 pg/mL, 111 pg/mL and 138 pg/mL, respectively. Supplementation of *Lactobacillus plantarum* with both levels of aflatoxins reduced the serum interleukin concentrations. The mean concentrations of interleukins IL-2, IL-4 and IL-6 in the chickens of the AF1LP group were 170.66 pg/mL, 70.33 pg/mL and 100 pg/mL, while AF2LP showed 199 pg/mL, 72.33 pg/mL and 109 pg/mL, respectively. IL-2, IL-4 and IL-6 concentrations in the serum were significantly lower in the AF1LP and AF2LP groups when compared with the AF1 and AF2 groups. 

### 2.5. Gross Pathology

**Figure 4:**
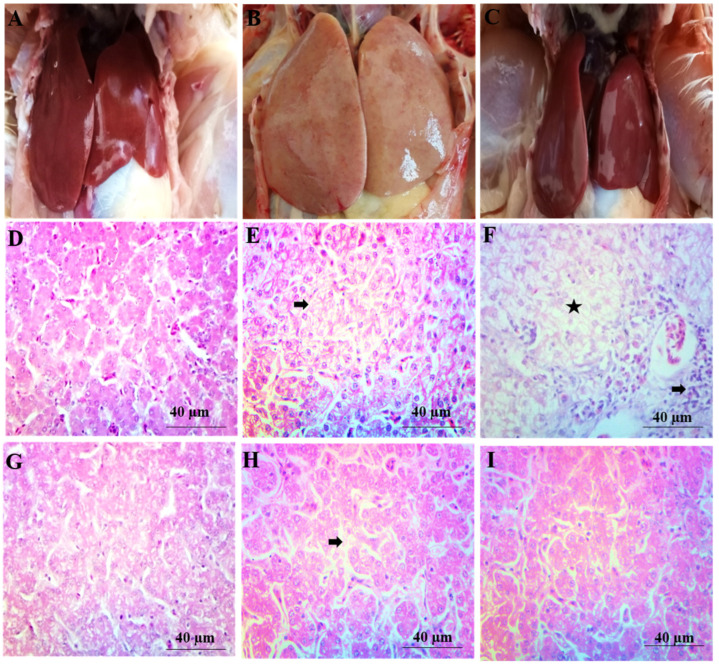
Gross and microscopic changes in the liver of broilers treated with aflatoxins and *Lactobacillus plantarum*. (**A**) Photograph of liver of control group showing the normal liver; (**B**) Photograph of liver of AF2 treated with aflatoxins (600 μg/kg) showing enlarged, swollen and pale liver; (**C**) Photograph of liver of group AF1LP treated with aflatoxins (300 µg/kg) and LP (1 × 10^8^ cfu/kg) showing almost normal liver; Microscopic changes in the hepatic parenchyma of the chicks fed aflatoxins and *Lactobacillus plantarum*. (**D**) Photomicrograph of liver of control group showing the normal hepatic parenchyma (400×); (**E**) Photomicrograph of liver of AF1 treated with aflatoxins (300 µg/kg) showing mild degenerative changes (arrow) in hepatic parenchyma (400×); (**F**) Photomicrograph of liver of group AF2 showing severe cellular swelling (star) and cellular infiltration (arrow) (400×); (**G**) Photomicrograph of liver of group AF1LP treated with aflatoxins (300 µg/kg) and LP (1 × 10^8^ cfu/kg) showing nearly normal hepatocytes and sinusoidal spaces (400×); (**H**) Photomicrograph of liver of group AF2LP treated with aflatoxins (300 µg/kg) and LP (1 × 10^8^ cfu/kg) showing less dilatation of sinusoidal spaces (arrow) as compared to AF2 group (400×); (**I**) Photomicrograph of liver of group LP (1 × 10^8^ cfu/kg) showing normal hepatic parenchyma (400×).

**Figure 5:**
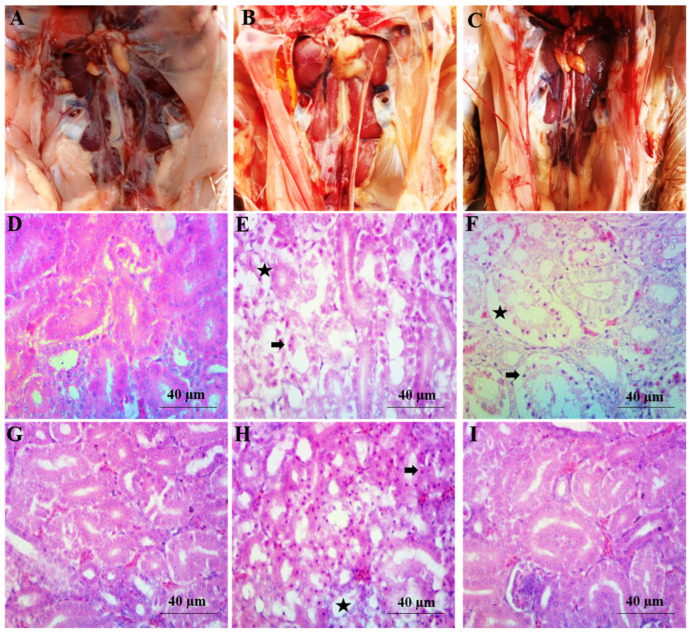
Gross and microscopic changes in the renal parenchyma of the chicks fed aflatoxins and *Lactobacillus plantarum*. (**A**) Photograph of kidney of control group showing normal kidneys; (**B**) Photograph of kidney of AF1 treated with aflatoxins (300 µg/kg) showing swollen kidney; (**C**) Photograph of kidney of AF1LP treated with aflatoxins (300 µg/kg) and LP (1 × 10^8^ cfu/kg) showing nearly normal kidneys; (**D**) Photomicrograph of kidney of control group showing normal renal parenchyma (400×); (**E**) Photomicrograph of kidney of AF1 treated with aflatoxins (300 µg/kg) showing necrosis (arrow) and loss of renal tubular epithelium (star) (400×); (**F**) Photomicrograph of kidney of the AF2 treated with aflatoxins (600 µg/kg) showing severe tubular degeneration (star) and necrotic changes (arrow) in renal tubular epithelium (400×); (**G**) Photomicrograph of kidney of AF1LP group treated with aflatoxins (300 µg/kg) and LP (1 × 10^8^ cfu/kg) showing almost normal renal parenchyma (400×); (**H**) Photomicrograph of kidney of AF1LP group treated with aflatoxins (600 µg/kg) and LP (1 × 10^8^ cfu/kg) showing tubular epithelial degeneration (star) and pyknotic nuclei (arrow) (400×); (**I**) Photomicrograph of kidney oLP group (1 × 10^8^ cfu/kg) showing normal renal parenchyma (400×).

The gross and microscopic changes in the liver and kidney are shown in Figure 4 and Figure 5 respectively.

The appearance of the liver and kidney was normal, and no gross lesions were observed on their surfaces in the BD and LP groups. The texture and color of the liver and kidney were normal, and the kidneys were present in their bony socket. However, the kidneys of AF1 were mildly bulging out from the bony sockets, and the liver of this group was also enlarged. The bulging of the kidneys was more prominent from the bony socket, and the liver was more enlarged in the AF2 group. On the surface of the kidney and liver, mild hemorrhages were present in AF1, while these changes were severe in AF2. The lesions observed in the kidney and liver of group AF2 were similar to group AF1 but with high intensity. Friable and darker liver with enlarged size was observed, and the kidneys were more bulged out from their sockets. Hemorrhages both petechial and ecchymotic were also observed in the kidney and liver. In the birds of AF1LP, the liver and kidney were normal. The liver of group D was normal, while in group AF2LP, the liver was slightly enlarged. The gross picture of the liver and kidney appears almost normal in AF1LP when compared with the AF1 group, while partial amelioration was observed in the AF2LP group when compared with the AF2 group. 

### 2.6. Histopathological Examinations

Arrangement of hepatic cords was normal in hepatic parenchyma in the control group. A similar pattern was also observed in group BDLP in which *Lactobacillus plantarum* was fed alone. The cytoplasm of group B showed some pyknotic nuclei, and mild degree degeneration was observed in hepatocytes. The group which was treated with *Lactobacillus plantarum* showed a similar picture. The cytoplasm of group B showed a mild degree of vacuolar degeneration (AF1). Liver of AF2 treated with aflatoxins (600 µg/kg of feed) showed degenerative changes in hepatic parenchyma and cellular infiltration around blood vessel. Almost normal liver parenchyma was observed in the AF1LP group (aflatoxins 300 µg/kg + *Lactobacillus plantarum* 1 × 10^8^ cfu/kg). Hepatic parenchyma of AF2LP group treated with aflatoxins (600 µg/kg) and LP (1 × 10^8^ cfu/kg) showed vacuolar changes in hepatocytes along with mildly congested sinusoidal spaces. Renal parenchyma of the control group was normal, while the necrosis loss of tubular epithelium was observed in the renal parenchyma of the AF1 and AF groups. The AF2LP group also showed necrotic and degenerative changes, while the AF1LP group showed almost normal renal parental parenchyma. The microscopic picture of liver and kidney of AF1LP showed almost normal parenchyma when compared with the AF1 group, while AF2LP showed mild-to-moderate microscopic changes in the hepatic and renal parenchyma. 

### 2.7. Residues of Aflatoxins in Liver and Kidney

**Figure 6:**
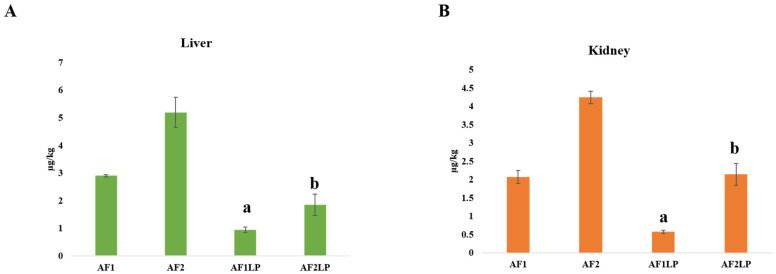
Residues of aflatoxins in the liver (**A**) and kidney (**B**). Vertical error bars with letters a and b are significantly different from AF1 and AF2 respectively. **Treatments:** BD = Basal diet; AF1 = Aflatoxins 300 µg/kg of feed; AF2 = Aflatoxins 600 µg/kg of feed; AF1LP = Aflatoxins 300 µg/kg of feed + *Lactobacillus plantarum* 1 × 10^8^ CFU; AF2LP = Aflatoxins 600 µg/kg of feed + *Lactobacillus plantarum* 1 × 10^8^ CFU; BDLP = Basal diet + *Lactobacillus plantarum* 1 × 10^8^ CFU.

Residues of aflatoxins in the liver and kidney are presented in Figure 6. 

Aflatoxins residues were not found in the liver and kidney of the BD and LP groups, in which a basal diet and *Lactobacillus plantarum* were fed to the chickens, respectively. The concentration of aflatoxins in the liver of chicks fed aflatoxins 300 and 600 µg/kg was 2.9 µg and 5.19 µg, respectively. The concentration of aflatoxins in the liver of chickens fed the diets contaminated with both doses of aflatoxins (300 and 600 µg/kg) and supplemented with *Lactobacillus plantarum* was 0.94 µg and 1.85 µg, respectively. In chickens receiving the diet with (300 and 600 µg/kg) aflatoxins, the mean concentration of aflatoxins was 2.07 µg and 4.24 µg, respectively, whereas when these chickens were supplemented with *Lactobacillus plantarum*, it was 0.57 µg and 2.14 µg lower, respectively. The differences between those supplemented and not supplemented were statistically significant. A significant reduction was observed in the aflatoxins residues in liver and kidney of the groups AF1LP and AF2LP when compared with the AF1 and AF2 groups. 

## 3. Discussion

Contamination of poultry feed with aflatoxins is a serious issue in the poultry industry, and its causes substantial loses to farmers. Producers face severe economic losses due to the sub-lethal but toxic effects of aflatoxins. In the present study, the efficacy of *Lactobacillus plantarum* imported from TISTR (Thailand Institute of Scientific and Technological Research) was evaluated to prevent the toxicological effects of aflatoxins in the broiler feed stuff. The performance and biochemical parameters, macroscopic and microscopic changes along with hematological alterations were determined. 

Broiler performance was affected by aflatoxins, and supplementation of *Lactobacillus plantarum* (1 × 10^8^ cfu/kg) ameliorates the pathological alterations when used with the first level of aflatoxins (300 µg/kg), but partial amelioration was observed when *Lactobacillus plantarum* was used with the second level of aflatoxins (600 µg/kg). 

The broilers which were treated with aflatoxins showed decreased body weight and feed intake. The reduction in feed intake and body weight might be due to less absorption of feed from the intestine, as aflatoxins damage the intestinal wall [15]. The other possible mechanism that can cause reduction in the feed intake and body weight due to aflatoxicosis is the lower production of digestive enzymes from pancreas. Our findings are in line with the results of Rodricks and Stoloff, who observed a 5–10% reduction in body weight when the chickens were fed with 2.5 mg aflatoxins per kg [16]. Hamilton et al. reported an 81 g reduction in body weight gain when chickens ingested 2.5 mg aflatoxins/kg of feed, and with 5 mg aflatoxins, that reduction was 161 g [17]. When the mixture of aflatoxins was fed to chickens at a dose rate of 3.5 mg, a reduction in the body weight gain was observed [18]. The incorporation of aflatoxins in chickens at a dose rate of 2 mg/kg resulted in reduction in body weight gain by 20–30% [19]. Similar results were also reported by [20,21,22]. When the *Lactobacillus plantarum* was used in combination with aflatoxins, it improved the feed intake and body weight. However, that mitigation was almost complete in the AF1LP group, and partial mitigation was observed in the AF2LP group. *Lactobacillus plantarum* has the ability to improve the feed intake and body weight against 300 µg aflatoxins, and it partially reduced feed intake and body weight against 600 µg aflatoxins. *Lactobacillus plantarum* bound with aflatoxins and removed the aflatoxins from the intestine, which leads to low levels of aflatoxins in the intestine, which might be a possible mechanism in improving the feed intake and body weight of broilers. The feed intake was improved in the group fed *Lactobacillus plantarum* alone (BDLP) group, and such results support the findings of the experimental study of [23], which reported that LP is a good agent to improve the performance of broiler chicks. Similar results were reported by [24] when he used a mixture of probiotics in the feed to control the aflatoxins toxicities in broilers.

Aflatoxins accumulate in liver and cause different pathological effects in hepatic parenchyma, and one of these effects is the absolute weight of liver [25,26,27,28,29,30]. Aflatoxins increased the weight of liver and kidney. Quezada et al. fed the 2 mg/kg aflatoxins and observed absolute liver and kidney weights [19]. Smith treated chickens with 3.5 mg/kg of feed aflatoxins and observed liver and kidney weights [18]. When the *Lactobacillus plantarum* was given to the broilers intoxicated with aflatoxins, it returned the absolute weight of the liver to normal. The reduction in the absolute weight was more prominent in the AF1LP group as compared to the AF2LP group. A similar pattern in absolute weight of kidney was observed as in liver. 

In the macroscopic examination, liver and kidney of broilers fed a basal diet showed normal color, while the liver of the aflatoxins-contaminated broilers showed a typical color (pale yellow). The present results agree with the authors who observed microscopic lesions in the liver and kidney of broilers fed aflatoxins-contaminated diets [27,28,29,30,31]. The broilers which were fed a basal diet and *Lactobacillus plantarum* showed no microscopic lesions. 

The concentrations of hepatic and renal biomarkers were increased in the broilers treated with aflatoxins, and that enhancement was achieved in a dose-dependent manner. The mean concentrations of ALP and of the AF1 and AF2 groups were 80.63 and 105.08 U/L, respectively, which were significantly higher than normal. The average values of ALT in the AF1 and AF2 groups were 30.94 and 34.74, respectively, while GGT showed 25.06 and 32.36, respectively. The serum concentrations of LDH and AST were also increased in aflatoxins-intoxicated groups. The mean concentration of urea and creatinine in the AF1 and AF2 groups were 31.52 and 39.45, respectively, while higher values of urea and creatinine were observed in the AF2 group. The supplementation of *Lactobacillus plantarum* returned these concentrations to the normal in AF1LP, while partial amelioration was observed in AF2LP. The main target organ for the aflatoxins is the liver followed by the kidney. When aflatoxins reaches the liver and kidney via blood circulation, they cause damage to liver and kidney tissues and lead to higher concentrations of hepatic and renal biomarkers. Aflatoxins reduced the level of serum proteins. Such results are in the accordance with the findings reported earlier by chen et al., Hassan et al., and Rathod et al. [32,33,34,35]. *Lactobacillus plantarum* ameliorated these pathological alterations when used in combination with aflatoxins. Amelioration was complete in AF1LP, and partial amelioration was observed in the AF2LP group. 

Regarding hematological parameters, the erythrocyte count, leukocyte count, hematocrit and hemoglobin concentration were significantly reduced. The reduction in the RBCs was 28% in AF1 and 36% in AF2, while WBCs were reduced by 24.22% and 47.82% in AF1 and AF2, respectively. Similar trends were observed in hemoglobin and hematocrit concentrations. *Lactobacillus plantarum* supplementation restored the red blood cells and white blood cells values in the AF1LP and AF2LP groups; however, that restoration was almost complete in the AF1LP group, and partial restoration was observed in the AF2LP group [29] also reported the decrease in RBC, WBC, Hb and MCV in birds fed 1.5 mg/kg of AFB1 in feed in broiler birds. Our results were also in argument with [26,34], whereby a reduction in these parameters being suggestive of anemia might be due to the adverse effects of AF on bone marrow, resulting in the suppression of hematocrit system, resulting in the reduced production of these cells. A decrease in the leucocyte count might have occurred due to the stress condition exhibited by AF supplementation to chicks. Supplementation of LP with AF1 significantly improved the hematological parameters, while that improvement was partial when LP was given with AF2. Anchang et al., 2016, reported a similar increase in RBCs count, WBCs count, hematocrit value and hemoglobin concentration in rat supplemented with probiotics in the feed [1].

Aflatoxins reduced the TAC by 49.08% and 58.24% in the AF1 and AF2 groups, respectively. Similar findings were reported by Ali et al., 2021, namely, that feeding of aflatoxins to the chickens for 42 days significantly reduced the TAC values in the serum [35]. Plantarum supplementation restored the TAC concentration in the AF1LP group, while AF2LP showed partial restoration. In the BDLP group, a 42.49% higher TAC value was observed, which was significantly high as compared to the control. 

In the aflatoxins treated groups (AF1 and AF2), significantly high TOS values were observed as compared to the control, while the BDLP group showed significantly lower TOS values. Among the combination groups, the TOS value was nearly normal in AF1LP, while AF2LP showed a significantly high value as compared to the control, but that was lower than AF2. Previously, Ali et al., 2021, reported that the feeding of aflatoxins-contaminated diets for 42 days significantly increased the serum TOS concentrations in the broilers [35]. 

Interleukins are components of the immune system, and they play an important role in inflammatory status, and when there is any disturbance in the level of interleukins, the pathological interventions occur. In the present study, the chickens which were fed with aflatoxins had significantly increased concentrations of interleukins IL-2, IL-4 and IL-6. The highest interleukins concentrations were observed in the AF2LP group followed by AF1LP. Similar findings were observed by Lai et al., 2022, who fed 200 µg/kg of feed aflatoxins to the chickens for 42 days and observed the high concentrations of interleukin-1β (IL-1β) and IL-6 [36]. The supplementation of *Lactobacillus plantarum* returned the interleukins values to nearly normal in the AF1LP group, while in the AF2LP group, partial amelioration was observed. In the BD and LP groups, no changes were observed in the concentrations of interleukins. Our findings are in line with Wang et al., 2015, who reported that *Lactobacillus plantarum* improved the immune performance of broilers [37].

In the chickens fed 300 and 600 µg/kg aflatoxins, the concentration of aflatoxins in the liver was 2.9 µg and 5.19 µg, and in the kidney it was 2.07 µg and 4.24 µg, respectively. Tejada-Castañeda et al. reported that when chickens were fed with 2 µg of AFB1/kg of feed for 21 days, the concentration of the AFB1 in the liver was 1–1.2 µg/kg, and in the kidney it was 0.8 µg [38,39]. *Bacillus subtilis* lowered the residues of aflatoxins in the liver by 42–97% in the broilers [39]. Yet, no report is available regarding the lowering of the concentration of aflatoxins in liver and kidney by using *Lactobacillus plantarum*. In our study the concentration of aflatoxins in the liver of chickens fed aflatoxins 300 and 600 µg/kg aflatoxins was reduced in liver and kidney compared to the birds fed contaminated diets but not supplemented with *Lactobacillus plantarum*. 

## 4. Conclusions

From this study, it is concluded that *Lactobacillus plantarum* has the ability to mitigate the histopathological, hematological, toxicopathological and immunological changes induced by 300 µg AF; however, that protection was partial when LP was administered with 600 µg. Though the results in this case were improved with this administration when compared with the individual AF group, complete amelioration of the alterations was not observed. Further studies are still needed to decide the exact ratio of AF:LP that causes such ameliorations. This study cleared that the use of *Lactobacillus plantarum* can prevent the toxic effects of AF in broiler chicks.

## 5. Materials and Methods

### 5.1. Ethical Approval

The present study has been conducted in compliance with the ethical standards of Institutional bioethics committee (IBC), University of Agriculture, Faisalabad, Pakistan. Ethical approval code was D. No. 3741/ ORIC UAF, dated 12 June 2019. This study was compiled with all the relevant legislations and experimental research was approved by the IBC on 12 June 2019. The experimental birds were kept under standard animal housing conditions. At the end of the experiment, six birds from the each group were slaughtered/killed by cervical dislocation, i.e., according to the standard procedure approved by the IBC, University of Agriculture, Faisalabad, Pakistan.

### 5.2. Aflatoxins Production and Propagation of Lactobacillus plantarum

Aflatoxins were produced by inoculating the culture of *Aspergillus flavus* (NRRL 6050; CECT 2948 on the basmati rice (100 g) in Erlenmeyer flask. The rice in the flask were soaked with distilled water (50 mL) for 2 h and autoclaved, at 121 °C, for 20 min prior to inoculation with fungal spores suspension (Hussain et al., 2008). After 7 days of incubation (28 degree Celsius) in the dark, aflatoxins were extracted from the rice by using chloroform and quantified according to the method of Association of Official Analytical Chemists (AOAC), method #990.33, using high-performance liquid chromatography (HPLC-FD), equipped with fluorescent detector. *Lactobacillus plantarum* was procured from the TISTR (Thailand Institute of Scientific and Technological Research). *Lactobacillus plantarum* was activated on MRS agar plate then mixed and incubated in liquid MRS medium for 24 h. After this, LP liquid solution was diluted until the concentration of bacteria reached to required concentration. 

### 5.3. Plan of Study

One hundred and eighty (180) healthy A grade chicks were purchased from the hatchery and divided into six groups. Trial was conducted by using different fractions of AF alone and in combination with *Lactobacillus plantarum* (LP) in chicks feed. Aflatoxins were produced in the lab and fed to chicks at the dose rate of 300 and 600 µg/kg of body weight. Ad libitum feed and water were offered to the chicks during the whole experimental period. Duration of the experiment was 6 weeks. Layout of the experiment has shown in Table 4.

### 5.4. Parameters Studied

#### 5.4.1. Performance Parameters

Weights of the chicks were recorded weekly. Six birds from each group were sacrificed at end of trail; absolute organ weight of liver and kidney was determined, and their relative weights were calculated as percentage of body weight.

$$\mathrm{Relative}\;\mathrm{organ}\;\mathrm{weight}\;\left(\%\right)=\frac{\mathrm{Organ}\;\mathrm{Weight}}{\mathrm{Body}\;\mathrm{weight}}\times 100$$

#### 5.4.2. Gross and Microscopic Pathology

Birds were sacrificed, and macroscopic lesions on kidney and liver were noticed. These organs were fixed in 10% neutral-buffered formalin and processed for histopathological examination following the method of Bancroft and Gamble [40]. Morphological alterations were observed under light microscope.

#### 5.4.3. Hematological Examination

Hematological parameters were estimated as described by Benjamin [41].

#### 5.4.4. Serum Biochemistry

Biuret method and bromocresol green dye binding method were used to determine the serum proteins and serum albumen (Davies et al., 1984). Commercially available kits were used for examination of ALT, AST, GGT, LDH, ALP, creatinine and blood urea (alanine assay kit catalogue MAKOO1, AST assay activity kit catalogue MAKO55, GGT assay kit catalogue MAK089, KDH activity assay kit catalogue MAK066, ALP activity kit AP0100, creatinine assay kit MAK080, and urea assay kit MAK006, Merck Germany).

#### 5.4.5. Serum Interleukins

Interleukin (IL-2, IL4 and IL-6) were analyzed using the ELISA kits. (Becton, Dickinson and Company, Franklin Lakes, NJ, USA) following the manufacturer’s instructions.

#### 5.4.6. Serum Total Oxidative Stress (TOS; μmol/L)

Eppendorfs were arranged in two and serum was dispensed in these tubes. Then, 225 µL of R1 were added in the each row for estimating the serum TOS. The samples which were kept in the first row their absorption was determined at 800 and 560 nm using the spectrophotometer (Thermoscientific Multiscan G0, Thermo Fisher). Serum samples which were placed in the second row were dispensed with R2 (11 µL) and absorption was observed at 560 nm and 800 nm.

Following formula was used to assess TOS.

Total oxidative stress (TOS) μmol/L = Absorbance/0.85 − 0.204

#### 5.4.7. Serum Total Antioxidant Capacity (TAC; mmol/L)

Eppendorfs were arranged in two rows and serum samples were taken in these tubes. After this, 200 μL of R1 were added in each serum sample, and the absorbance was observed at 560 nm. Then, R2 (20 μL) was added in second row and absorbance was measured at 660 nm by using a spectrophotometer.

Total antioxidant capacity was estimated by following formula:

Total antioxidant capacity (TAC) = (Absorbance − 0.947)/0.2829

#### 5.4.8. Residues of Aflatoxins in Liver and Kidney

Aflatoxin concentration in the liver and kidney was determined by enzyme linked immunosorbent assay (ELISA). Commercial ELISA kit AgraQuant Aflatoxin (Romer Lab Diagnostic, Singapore) was used to detect the aflatoxin residues in the liver and kidney. The procedure mentioned in the AgraQuant Aflatoxin kit manual was followed to determine the residues of aflatoxins.

#### 5.4.9. Statistical Analysis

Analysis of variance (ANOVA) was used to analyze the data, and means were compared by Tukey’s, using SAS University Edition online software SAS stat 15.1 [42].

## Figures and Tables

**Table 1:** Feed intake broiler birds fed aflatoxins and *Lactobacillus plantarum* in different combinations (Mean ± SD).

Groups	Week 1	Week 2	Week 3	Week 4	Week 5	Week 6
**BD**	57.41 ± 14.12 a	97.76 ± 12.44 a	128.13 ± 18.76 b	156.49 ± 6.24 b	199.31 ± 9.56 b	230.53 ± 11.17 b
**AF1**	51.14 ± 13.23 a	84.69 ± 9.56 b	103.42 ± 14.87 c	145.17 ± 7.84 c	182.74 ± 14.53 c	198.35 ± 14.27 c
**AF2**	45.15 ± 11.23 a	75.09 ± 8.57 c	92.77 ± 16.23 d	136.73 ± 5.53 d	166.14 ± 8.67 d	174.46 ± 12.54 d
**AF1LP**	56.52 ± 14.81 a	98.27 ± 9.32 a	127.45 ± 17.63 b	155.17 ± 5.45 b	197.73 ± 8.13 b	227.48 ± 9.21 b
**AF2LP**	54.84 ± 13.73 a	85.65 ± 12.74 b	116.34 ± 28.67 c	144.32 ± 7.51 c	179.78 ± 7.63 c	187.16 ± 13.23 c
**BDLP**	60.47 ± 13.73 a	99.11 ± 5.87 a	148.74 ± 15.83 a	172.63 ± 5.87 a	216.74 ± 11.73 a	252.84 ± 14.87 a

Values with different letters in each column are significantly different (*p* ≤ 0.05). **Treatments:** BD = Basal diet; AF1 = Aflatoxins 300 µg/kg of feed; AF2 = Aflatoxins 600 µg/kg of feed; AF1LP = Aflatoxins 300 µg/kg of feed + *Lactobacillus plantarum* 1 × 10^8^ CFU; AF2LP = Aflatoxins 600 µg/kg of feed + *Lactobacillus plantarum* 1 × 10^8^ CFU; BDLP = Basal diet + *Lactobacillus plantarum* 1 × 10^8^ CFU.

**Table 2:** Weekly body weights of groups fed aflatoxins and *Lactobacillus plantarum* alone and in combination (Mean ± SD).

Group	Initial Weight	Week 1	Week 2	Week 3	Week 4	Week 5	Week 6
**BD**	40 ± 4.30 a	86.23 ± 6.57 a	237.35 ± 18.52 a	861.83 ± 30.14 b	1242.74 ± 44.51 b	1728.51 ± 54.14 b	2012.53 ± 66.49 b
**AF1**	45 ± 3.70 a	83.39 ± 10.83 a	214.49 ± 12.67 c	799.23 ± 40.15 c	1031.69 ± 53.23 c	1459.23 ± 94.67 c	1679.48 ± 64.43 c
**AF2**	47 ± 3.50 a	74.74 ± 11.57 a	165.47 ± 22.73 d	677.09 ± 55.34 d	880.79 ± 51.23 d	1322.23 ± 57.57 d	1559.21 ± 37.54 d
**AF1LP**	39 ± 5.3 a	72.15 ± 6.13 a	229.63 ± 13.47 b	859.48 ± 30.83 b	1227.12 ± 38.19 b	1702.27 ± 81.51 b	1952.34 ± 51.43 b
**AF2LP**	51 ± 3.90 a	71.11 ± 5.38 a	212.49 ± 10.51 c	809.67 ± 30.52 c	1095.72 ± 81.53 c	1492.17 ± 79.84 c	1632.65 ± 71.86 c
**BDLP**	42 ± 4.31 a	78.23 ± 6.76 a	249.28 ± 10.64 a	902.0 ± 33.1 a	1368.63 ± 21.56 a	1787.16 ± 46.23 a	2087.29 ± 43.68 a

Values with different letters in each column are significantly different (*p* ≤ 0.05). **Treatments:** BD = Basal diet; AF1 = Aflatoxins 300 µg/kg of feed; AF2 = Aflatoxins 600 µg/kg of feed; AF1LP = Aflatoxins 300 µg/kg of feed + *Lactobacillus plantarum* 1 × 10^8^ CFU; AF2LP = Aflatoxins 600 µg/kg of feed + *Lactobacillus plantarum* 1 × 10^8^ CFU; BDLP = Basal diet + *Lactobacillus plantarum* 1 × 10^8^ CFU.

**Table 3:** Relative weight of different organs of the birds fed aflatoxins and *Lactobacillus plantarum* alone and in combination (mean ± SD).

Group	Liver	Kidney	Spleen	Bursa	Thymus
**BD**	3.20 ± 0.16 d	0.66 ± 0.06 d	0.16 ± 0.03 a	0.19 ± 0.01 b	0.40 ± 0.02 b
**AF1**	5.62 ± 0.38 b	1.40 ± 0.06 b	0.14 ± 0.01 a	0.11 ± 0.01 c	0.27 ± 0.01 c
**AF2**	6.50 ± 0.32 a	1.85 ± 0.07 a	0.12 ± 0.02 a	0.06 ± 0.01 d	0.21 ± 0.02 d
**AF1LP**	3.26 ± 0.15 d	0.69 ± 0.07 d	0.15 ± 0.03 a	0.18 ± 0.01 b	0.39 ± 0.01 b
**AF2LP**	4.80 ± 0.33 c	0.95 ± 0.08 c	0.15 ± 0.02 a	0.08 ± 0.03 c	0.23 ± 0.03 cd
**BDLP**	3.21 ± 0.22 d	0.59 ± 0.04 d	0.17 ± 0.01 a	0.24 ± 0.01 a	0.45 ± 0.01 a

Values with different letters in each column are significantly different (*p* ≤ 0.05). **Treatments:** BD = Basal diet; AF1 = Aflatoxins 300 µg/kg of feed; AF2 = Aflatoxins 600 µg/kg of feed; AF1LP = Aflatoxins 300 µg/kg of feed + *Lactobacillus plantarum* 1 × 10^8^ CFU; AF2LP = Aflatoxins 600 µg/kg of feed + *Lactobacillus plantarum* 1 × 10^8^ CFU; BDLP = Basal diet + *Lactobacillus plantarum* 1 × 10^8^ CFU.

**Table 4:** Experimental layout.

Group	No. of Birds	Treatment
BD	30	Control (Basal diet)
AF1	30	Aflatoxins 1st level (300 µg/kg of b.wt)
AF2	30	Aflatoxins 2nd level (600 µg/kg of b.wt)
AF1LP	30	Aflatoxins 1st level + *Lactobacillus plantarum*
AF2LP	30	Aflatoxins 2nd level + *Lactobacillus plantarum*
BDLP	30	*Lactobacillus plantarum* (1 × 10^8^ cfu/kg)

## Data Availability

The data presented in this study is available on the request from corresponding author.

## Abbreviations

AF	Aflatoxins
AF1	Aflatoxins 1st level
AF2	Aflatoxins 2nd level
LP	*Lactobacillus plantarum*
RBCs	Red blood cells
WBCs	White blood cells
Hb	Hemoglobin concentration
PCV	Packed cell volume
ALT	Alanine aminotransferase
AST	aspartate aminotransferase
GGT	Gamma glutamyle transferase
LDH	Lactate dehydrogenase
ALP	Alkaline phosphatase
